# Assessing the Quality of Care at Primary Health Care Level in Two Pilot Regions of Albania

**DOI:** 10.3389/fpubh.2021.747689

**Published:** 2021-12-22

**Authors:** Jasmina Saric, Sabine Kiefer, Altina Peshkatari, Kaspar Wyss

**Affiliations:** ^1^Swiss Centre for International Health, Swiss Tropical and Public Health Institute, Basel, Switzerland; ^2^University of Basel, Basel, Switzerland; ^3^HAP Center, Tirana, Albania

**Keywords:** Albania, quality of care, clinical observations, patient satisfaction, infrastructure

## Abstract

The quality of care (QoC) of primary health care (PHC) services in Albania faces challenges on multiple levels including governance, access, infrastructure and health care workers. In addition, there is a lack of trust in the latter. The Health for All Project (HAP) funded by the Swiss Agency for Development and Cooperation therefore aimed at enhancing the population's health by improving PHC services and implementing health promotion activities following a multi-strategic health system strengthening approach. The objective of this article is to compare QoC before and after the 4 years of project implementation. A cross-sectional study was implemented at 38 PHC facilities in urban and rural locations in the Diber and Fier regions of Albania in 2015 and in 2018. A survey measured the infrastructure of the different facilities, provider–patient interactions through clinical observation and patient satisfaction. During clinical observations, special attention was given to diabetes and hypertensive patients. Infrastructure scores improved from base- to endline with significant changes seen on national level and for rural facilities (*p* < 0.01). Facility infrastructure and overall cleanliness, hygiene and basic/essential medical equipment and supplies improved at endline, while for public accountability/transparency and guidelines and materials no significant change was observed. The overall clinical observation score increased at endline overall, in both areas and in rural and urban setting. However, infection prevention and control procedures and diabetes treatment still experienced relatively low levels of performance at endline. Patient satisfaction on PHC services is generally high and higher yet at endline. The changes observed in the 38 PHC facilities in two regions in Albania between 2015 and 2018 were overall positive with improvements seen at all three levels assessed, e.g., infrastructure, service provision and patient satisfaction. However, to gain overall improvements in the QoC and move toward a more efficient and sustainable health system requires continuous investments in infrastructure alongside interventions at the provider and user level.

## Introduction

Healthcare in the Soviet Union (1922–1991) was delivered by a state-run, centralized, integrated and hierarchically organized health-care model—the Semashko system—providing state-funded health care to all citizens. The system was widely adopted by the Soviet Union's satellite and aligned states, being perceived as a coherent and cost-effective model to cope with the medical necessities at the time. Indeed, the system allowed the former Soviet Union to pioneer something close to universal coverage of basic health-care services (1). However, since the post-Soviet transition, ex-Semashko states across Eastern Europe and northern Asia have been undergoing major health system reforms.

Albania, a formerly aligned state in Southeast Europe broke with the Soviet Union in 1961. While the country adopted aspects of the Semashko model, including the development of an extensive primary health care (PHC) during the 1960s (2), it shifted to prioritizing hospital care in the 1970s. However, sustained periods of economic and political isolation within Europe as well as within the former Communist group of countries (2, 3), lead to great challenges to reforming Albania's economic and social establishments, including healthcare. In 1990–1991 the economy of Albania and inevitably all other sectors collapsed, leading to the fall of the communist regime and paving the way to democratic reforms. By the late 90ies, most hospital services were stripped down to providing emergency care only, almost one third of the medical staff force was lost and drugs and medical equipment were looted. The healthcare crisis was exacerbated by the spill-over from the Kosovo war with the Federal Republic of Yugoslavia that drove thousands of Kosovo Albanians into the country, putting additional strains on an already inadequate national healthcare system (2). Despite those challenges, multiple reform cycles were implemented by the Albanian Ministry of Health (today's Ministry of Health and Social Protection) during the last three decades. In the same period, Albania progressed economically to halving national poverty and achieving upper-middle-income status (3).

As part of the healthcare reform, Albania has invested in improving national PHC services to better accommodate current and future population health needs, above all, the increasing strain from non-communicable diseases, urbanization and population aging (2, 4–7). Yet, PHC services remain challenged on the levels of health systems governance (e.g., lack of autonomy for PHC center managers), information sharing (e.g., lack of an integrated national health information system and a dysfunctional referral and follow-up system), healthcare workers (e.g., shortage of and inadequate training and/or positioning of health workers vis-à-vis PHC needs), access (e.g., long waiting times and travel distances) and hardware (i.e., infrastructure and diagnostic equipment). Those challenges are exacerbated by a lack of trust in PHC staff members by patients and secondary/tertiary care representatives (8, 9). Consequently, PHC is often being bypassed in favor of secondary or tertiary care, and sometimes, private service providers (10).

PHC is deemed the gateway for universal health coverage (UHC) ensuring that most essential interventions can be accessed equitably, prioritizing on prevention and promotion and minimizing out-of-pocket spending (11). However, while access to basic health care services has increased on a global level, quality of care (QoC) has lagged behind owing to the lack of investment and interest in this domain and the wider perception that quality naturally improves with the expansion of coverage (12). In recent years, it has been recognized that this expectation does not reflect the reality and that poor QoC may be a significant challenge to achieving UHC, irrespective of access (13) as well as a major driver of excess mortality (14). Peer reviewed information on QoC in Albania is scarce and fragmented drawing from six studies that revealed sub-standard QoC in maternal and newborn health care, acute myocardial infarction and NCD patient care in Albanian hospitals (15–17). Most recent work, using cross-sectional health facility surveys to identify the factors associated with the utilization of PCH services, demonstrated that QoC was among the most important influencing factors for choosing a type of health facility for both public and private health facility users (18, 19). The perception of PHC users vis-à-vis non-clinical QoC and its association with sociodemographic characteristics of patients and the type of health provider (private vs. public) was analyzed in a related study, using a facility-based survey (20). The perception of non-clinical QoC was found to be high and similar for both types of providers with highest scores reported for clear “communication” and “dignity.” The lowest scores were given to “promptness” and “coordination of care” required attention to meet patient's expectations on good QoC.

The Health for All Project (HAP) funded by the Swiss Agency for Development and Cooperation (SDC) aims at strengthening the health system in Albania with a focus on PHC (21). The first phase of the project was implemented in two Regions of Albania from January 2015 to March 2019 in 80 primary health centers. The overall goal of HAP was to enhance the population's health by improving PHC services and implementing health promotion activities following a multi-strategic “health system strengthening” approach that supports actors in the health system on the demand side and the supply side. This was achieved by running health promotion campaigns and educational activities for the former, by strengthening capacities of health professionals through training and education and by enhancing infrastructure and equipment.

The objective of the here presented work was to compare QoC before and after the four years of PHC strengthening in the two study regions in Albania.

## Methods

### Study Framework

The HAP project was implemented in two pilot regions in Albania, namely Diber in the north-east of the country with a population of 120,978 and Fier, located in south-west Albania with a population of 298,144. Diber is a mountainous, rural area relying largely on agricultural production, while Fier has access to the sea, an oil industry, is a producer of agriculture and remains predominantly rural. A range of activities was implemented to strengthen QoC specifically, e.g., regular continuous medical education (CME) schemes through peer groups and the introduction of “Nurse bags” and “Doctors bags” along with training on their use. Content wise the training for both peer groups and doctor/nurse bags focussed on technical aspects of patient treatment and in selected instances on patient-provider principles. In addition, a complete renovation of 16 health centers took place.

Two cross-sectional studies on the QoC in PHC centers were conducted; one at baseline in 2015 and another one in 2018 after 4 years of HAP implementation. Data were collected at three different levels, e.g., (i) the health facility, (ii) the health provider; and (iii) the patients. Criteria for inclusion of the health facilities in the survey required that (i) they be situated in rural and urban areas; (ii) have at least one medical doctor working at the facility; (iii) the facility offered the provision of care and prevention related to chronic diseases (e.g., diabetes mellitus, hypertension); and (iv) and that they were covered by HAP and project activities, namely CME. Criteria for the inclusion of the health providers of the selected facilities for provider–patient observations required that they were general practitioners/family doctors, that oral informed consent of the PHC service provider was given and that written informed consent of the patient, or his or her legal representative, was obtained prior to each clinical observation. The distinction between general practitioners and family doctors is blurry in Albania. General practitioners often describe themselves as family doctors since the number of generalists trained in the specialization of family medicines is very low in Albania. The self-classification is thus generally relating to general practitioners being contracted as family doctors.

Finally, criteria for the inclusion of patients exiting the selected facilities and receiving consultation required that patients were at least 18 years old or accompanied by a legal representative. Moreover, they had to have accessed health centers and have received consultation from a health provider for their own health issue or the health issue of the child that they accompanied. Written informed consent of the patient, or his or her legal representative, had to be obtained prior to the study confirming the voluntary participation and acknowledging the right to withdraw from the study at any point in time.

The same 38 primary care facilities were assessed during baseline and endline studies, while PHC providers observed during clinical practice and patients exiting the health service differed. Diber accounted for 20 of the facilities surveyed, whilst Fier accounted for the remaining 18. The surveyed facilities were located into 27 rural and 11 urban areas.

### Survey

The survey measured structural, process and outcome attributes thereby following the framework as laid out by Donabedian (22, 23). It included three questionnaires to assess the different dimensions of QoC: (i) the health facility level as a proxy for structural attributes; (ii) health provider level (process attributes); and (iii) patient satisfaction (outcome attributes). The questionnaire in the endline survey remained largely identical to the baseline survey. They included a mix of questions from the World Health Organization (WHO) Service Availability and Readiness Assessment (SARA) (24) and an electronic Tool to Improve Quality of Health Care developed within the “ACCESS” program supported by the Novartis Foundation for Sustainable Development (25). The questionnaires were adapted to the local Albanian context thereby taking into consideration the MoHSP (2014) “Basic Package of Services in Primary Health Care” and the existing treatment guidelines for specialist care (adjusted for family doctors). Some questions relating to HAP interventions, HAP infrastructure improvements (e.g., rehabilitation and equipping doctors and nurses) and HAP providing information corners and community participation in health promotion activities, were introduced. Other questions were amended to reflect changes in policy and updated guidelines (e.g., complaint mechanisms, updated the age of check-up program from 40–65 in baseline to 35–70 endline and list of essential medicines).

The infrastructure assessment and patient satisfaction were conducted as tablet-based interviews. Interviews were based on structured and closed questions in a questionnaire. The patient interactions were documented in the frame of structured observations according to treatment protocols for (i) the principles of clinical history and physical examination; (ii) infection prevention and control measures; (iii) diabetes treatment; (iv) hypertension treatment; and (v) all other treatments. However, it should be noted, that the protocols for (iii) and (iv) relate to specialist treatment protocols as the MoHSP had not published treatment protocols for PHC.

### Training and Pre-test

Interviewers were trained and received clear instructions on the data collection, specifically the conduct of observations for the clinical consultations. Nevertheless, variations between interviewers/observers cannot be completely excluded. All interviewers had medical or a public health background. The interviewers were informed about (i) the HAP; (ii) the aim and objectives of the survey; (iii) the data collection process and procedures; iv) the structure of the questionnaires; and (v) the use of portable electronic tablet devices. A pre-test was conducted at two rural health facilities in Fier, which were different from the sampled health centers, and they were supervised by the regional coordinator and two HAP staff. All interviewers gained experience in clinical observations and exit interviews. To conduct the infrastructural assessment, the interviewer group followed a HAP supervisor and a doctor/director in the health center who showed and explained the different medical instruments.

A similar questionnaire was used as in previous studies. In order to assure content validity the HAP team collected the interviewer feedback after the pre-test and a few adjustments were made to the wording and translation of questions and answer possibilities. In a few instances, additional clarifications and choices were added.

### Data Collection

Fieldwork for the baseline survey took place between April 2015 and May 2015 and for the endline survey between July and August 2018. Procedures were mostly the same for both surveys; in brief for the endline survey, 12 interviewers (8 for the baseline), organized in teams of two, were closely supervised and supported whilst they conducted data collection of the three dimensions of QoC in health centers. Patients were included consecutively. The number of patients included per day did align to the interview workload for one data collection team in one given facility. Data collection was done electronically using tablets and Open Data Kit (ODK) software was used for the questionnaire. Completed questionnaires were transferred to a server in Basel, Switzerland on the same day where an initial quality check was conducted. Sampling was done separately for each region, applying a clustered approach (i.e., one cluster equalled 1 day of data collection at a health facility) proportional to size using the number of facility visits in the year before each survey as a proxy for the size of the facility. In total, 27 clusters were applied in each region and larger facilities were sampled several times. These (larger) facilities, typically in urban areas, were visited on consecutive days but different doctors were observed for clinical consultations. Data collectors typically visited a facility and observed as many clinical consultations as possible during each sampling day. The infrastructure evaluation was done only for health centers and not for the health posts and conducted together with the head of the facility, or his or her closest representative, by the end of the working day. For the clinical observations, patient illnesses were categorized into “hypertension,” “diabetes,” and “other” since previous studies have demonstrated issues with sample size when specifying additional categories. “Other” was to include any other reason for a doctor visit, including vaccination, flu/cold and diarrhea and obtaining a prescription. Following a consecutive sampling approach, exit interviews were requested from all patients exiting the facility by the data collectors. The interview with patients exiting the facility was conducted in the yard or in some cases where suitable, in the large HC corridors, to allow for maximum privacy.

### Data Analysis

Summary cross-tables were created for each variable and stratified according to the regions and the locations. Potential significant differences, between baseline and endline, were identified using the Wilcoxon signed-rank test for the paired ordinal infrastructural assessments, the Rao-Scott corrected chi-squared test for categorical variables, and mixed linear regression for continuous variables originating from clinical observations and exit interviews. The latter are indices based on sets of questions or items about a specific topic, e.g., infection prevention and control. The value of the index is the percentage of questions answered correctly or items fulfilled. Questions or items not answered or not applicable were excluded from the calculations. Data was analyzed using Stata Statistical Software v16.1 (Stata Corporation; College Station, TX, USA).

## Results

### Infrastructural Assessment

An improvement of the overall average infrastructure score was observed between base- to endline (*p* < 0.001) across the study facilities. Assessing the average percentage infrastructure score for each of six sub-topics, (1, facility infrastructure and overall cleanliness; 2, hygiene; 3, public accountability/transparency; 4, guidelines and materials; 5, basic/essential medical equipment and supplies; and 6, medication and products), improvements at endline were found in all sub-topics but in the guidelines and materials: (1) *p* = 0.003; (2) *p* < 0.001; (3) *p* = 0.020; (4) *p* = 0.79; (5) *p* < 0.001; and (6) *p* = 0.002.

### Clinical Observations

#### Socio-Economic Profile of Doctors

Overall, 842 clinical observations were conducted during the endline survey thereof 354 in Diber and 488 in Fier (at baseline: Total 625; Diber 175; Fier 450). The average number of observations per facility was 22 (median 19; min. 2; max. 64) with a lower average in Diber than in Fier (18 vs. 27 respectively). Differences in the number of consultations between the two regions reflect the different utilization rate of health services. In Diber, 33% of observations were conducted in urban facilities compared with 53% in Fier (Table 1).

**Table 1:** Observations and socio-demographic attributes of doctors of clinical consultations.

	**Baseline**					**Endline**				
	**Diber** **% (n)**	**Fier** **% (n)**	**Rural % (n)**	**Urban % (n)**	**Total % (n)**	**Diber % (n)**	**Fier** **% (n)**	**Rural % (n)**	**Urban % (n)**	**Total % (n)**
Number of observations	28.0 (175)	72.0 (450)	40.2 (251)	59.8 (374)	100.0 (625)	42.0 (354)	58.0 (488)	55.0 (463)	45.0 (379)	100.0 (842)
**Reason for visit**										
Arterial hypertension	24.6 (43)	30.9 (139)	24.3 (61)	32.4 (121)	29.1 (182)	32.8 (116)	23.6 (115)	28.5 (132)	26.1 (99)	27.4 (231)
Diabetes	2.3 (4)	8.7 (39)	2.4 (6)	9.9 (37)	6.9% (43)	4.5 (16)	7.0 (34)	5.6 (26)	6.3 (24)	5.9 (50)
Other	73.1 (128)	60.4 (272)	73.3 (184)	57.8 (216)	64.0 (400)	62.7 (222)	69.4% (339)	68.9 (305)	67.5 (256)	66.6 (561)
Number of doctors that were observed	48.1 (25)	51.9 (27)	50.0 (26)	50.0 (26)	100.0 (52)	44.2 (38)	55.8% (48)	50.0 (43)	50.0 (43)	100.0 (86)
Female doctors observed	60.0 (15)	77.8 (21)	42.3 (11)	96.2 (25)	69.2 (36)	71.1 (27)	75.0 (36)	55.8 (24)	90.7 (39)	73.3 (63)
**Type of doctor (contracted)**										
Family doctor	4.2 (1)	3.7 (1)	-	7.7 (2)	3.9% (2)	44.7 (17)	37.5 (18)	48.8 (21)	32.6 (14)	40.7 (35)
General doctor	96.0 (24)	92.6 (25)	100.0 (26)	88.5 (23)	94.2 (49)	55.3 (21)	62.5 (30)	51.2 (22)	67.4 (29)	59.3 (51)
Specialist	0.0 (0)	3.7 (1)	-	3.9 (1)	1.9 (1)	0.0 (0)	0.0 (0)	0.0 (0)	0.0 (0)	0.0 (0)

Mostly, patients attended the facility for health reasons “other” than diabetes and hypertension (baseline 64%; endline 66%) followed by hypertension (baseline 29%; endline 28%) and diabetes (baseline 7%; endline 6%). Specifically, diabetes was more prevalent among observations in urban facilities. Among patients, 56% were female. Observations were done for 86 doctors during the endline (and 52 doctors during the baseline) with an average of 9 observations per doctor (min: 1; max. 36). Thus, the ranges of observations are similar to the baseline study. Doctors were mostly female (73%) and 59% were general doctors, 41% contracted as family doctors (baseline general doctors 94%, family doctors: 4%; specialists 2%).

#### Clinical Consultations Scores

The average clinical observation score improved between baseline and endline across the study facilities (*p* < 0.001), as assessed by mixed linear regression adjusting for district and urban/rural, with random effect for health facility.

Adherence to principles of good clinical practice and physical examination achieved improved and high scores at endline, compared with the baseline, across the study facilities. Seeing the client in privacy/ensuring confidentiality saw a particularly large increase of over 20 percentage points in the overall score from 70.4% at baseline to 92.6% at endline (Table 2). Greeting of the client and the polite closing of the consultation was adhered to in most instances, in both evaluations.

**Table 2:** Rating for adherence to the principles of history and physical examination by the medical doctor and for infection prevention and control.

	**Baseline**		**Endline**		
	**Total % (n)**	***N***	**Total % (n)**	***N***	***p*** **-val^a^**
**Adherence to principles of history and physical examination by the medical doctor**					
Greets the client	96.6 (604)	625	99.9 (841)	842	<0.001
Sees the client in privacy/confidentiality	70.4 (440)	625	92.6 (780)	842	<0.001
Makes the client comfortable^b^	84.5 (528)	625	98.3 (828)	842	<0.001
Asks the client about concerns, allows client to explain his/her health issue	88.5 (553)	625	97.6% (822)	842	0.003
Closed politely the consultation	96.0 (583)	607^d^	98.8 (768)	777^d^	0.099
**Infection prevention and control**					
Washed hands before the procedure including use of soap	4.8 (30)	625	12.1 (93)	625^d^	0.037
Applied proper decontamination procedures^c^	0.0	90^d^	12.2 (44)	362^d^	0.061
Put on gloves where required	2.8 (3)	107^d^	5.1 (13)	253^d^	0.57
Put on a mask where required	0.0 (0)	116^d^	3.6 (9)	251^d^	0.062

a *Rao-Scott corrected chi-squared test;*

b *e.g., seat offered;*

c *e.g., soaking contaminated instruments into a bucket with chlorine or any other disinfectant;*

d *nature of observation was deemed not applicable by observers for some consultations leading to less overall observations*.

Infection prevention and control procedures showed a trend for improvement between 2015 and 2018 for all procedures except for putting on gloves when required (Table 2). However, the proportion of health staff that routinely implements hygiene measures, albeit slightly improved, remained at a very low level at endline (3.6% put on a mask when required, 5.1% put on gloves when required and 12.1% washed hands before the procedure).

Slightly more clinical consultations with diabetes patients were reported during the endline (*n* = 50) compared with the baseline (*n* = 43). During the endline evaluation, the number of diabetic patients observed in rural and urban centers were almost the same (rural *n* = 26, urban *n* = 24) in contrast to the baseline assessment, where observations in rural areas were six times lower than in urban settings (rural *n* = 6; urban *n* = 37). An overall improvement was found between base- and endline, with regard to treatment of diabetes patients by (i) asking questions (e.g., about adherence to treatment, health complaints, risk factors; (ii) conducting examinations (e.g., checks on blood pressure and eyes); and (iii) give advice, explanations or instructions (e.g., regarding follow-up visits, diagnosis, prescribed medicines) (*p* = 0.013) (Table 3). However, of the sub-categories, ‘giving advice, explaining and instructing’ was the only one that notably improved between base- and endline (*p* = 0.001).

**Table 3:** Clinical observation score—average achieved % out of all items for diabetes, hypertension and “other illnesses.”

	**Baseline**		**Endline**		
	**Average achieved %**	***N***	**Average achieved %**	***N***	***p*** **-val^a^**
**Out of all diabetes items**					
Asks questions	23.6	43	38.5	50	0.24
Conducts examination	10.9	43	21.8	50	0.59
Advices, explains, and instructs	25.1	43	51.0	50	0.001
Overall	22.3	43	42.4	50	0.013
**Out of all hypertension items**					
Asks questions	24.2	182	46.3	231	<0.001
Conducts examination	17.8	182	26.4	231	0.004
Advices, explains, and instructs	38.0	182	59.8	231	<0.001
Overall	29.4	182	47.4	231	<0.001
**Out of all other illnesses**					
Asks questions	71.1	400	86.0	561	<0.001
Conducts examination	59.7	400	82.1	561	<0.001
Advices, explains, and instructs	51.6	400	74.9	561	<0.001
Overall	63.5	400	80.2	561	<0.001

a *Mixed linear regression adjusting for district and urban/rural, with random effect for health facility*.

A total of 231 clinical hypertension consultations were observed at endline (*N* = 116 in Diber and *N* = 115 in Fier) and 182 at baseline (Diber *n* = 43; Fier *n* = 139). Of the endline observations 132 took place in rural (57%) and 99 in urban facilities (43%). A significant improvement was found in the scores for hypertension treatment where an overall increase from baseline to endline was observed (*p* < 0.001) (Table 3). The average scores in both evaluations were best for “giving advice” (baseline 38%; endline 60%) followed by “asking questions” (baseline 24%; endline 46%). All three sub-categories saw clear improvement between base- and endline (*p* < 0.01).

A total of 561 patient consultations were recorded during the endline survey for diseases other than diabetes or hypertension. Of these, 40% (*n* = 222) were conducted in Diber (baseline 32%; *n* = 128) and 60% (*n* = 339) in Fier (baseline 68%; *n* = 272). During the base- and endline surveys about half of the observations were conducted in rural locations (baseline 46%; endline 54%). Overall, consultations of patients for “other diseases” achieve higher scores than for diabetes and hypertension (Table 3). With regard to the average score on “other disease” treatment, a significant increase of observed between the base- and endline (*p* < 0.001). Asking questions improved from 71% from the baseline to 86% during the endline. Examinations were provided as required during 82% of cases in the endline compared to 60% of cases during the baseline. Advice and explanations achieved average scores of 75% during the endline and 52% during the baseline. Improvements at endline were achieved in each sub-category (*p* < 0.001).

### Exit Interviews

#### Respondents Socio-Economic Profile

Of 870 eligible patients exiting the health facilities during the endline survey, 776 patients participated in the survey resulting in a response rate of 89% (baseline: *n* = 706 of 769; response rate: 92%). Out of 776 conducted 325 (41.9%) were in Diber and 451 (58.1%) in Fier region. During the baseline, a larger difference was found between the two areas, i.e., 25.9% in Diber and 74.1% in Fier (Table 4). Similarly, a shift was observed toward less interviews being conducted in urban health centers at endline compared with the baseline (endline: 46.6%; baseline: 66.7%). The sample consists of 439 (57.0%) women and an average age of respondents of 51.6 years (min. 0 years, max. 86 years; median: 56 years). Respondents most commonly had about 8/9 years or 12 years of school education. Participants were most commonly pensioners, followed by being unemployed or a housewife. About 23.8% of participants benefited from economic or social aid at the time (baseline: 15%) and 4.5% belong to an ethnic or linguistic minority (baseline 3%).

**Table 4:** Socio-demographic attributes among respondents of exit interviews.

	**Baseline**					**Endline**				
	**Diber % (n)**	**Fier** **% (n)**	**Rural % (n)**	**Urban % (n)**	**Total % (n)**	**Diber % (n)**	**Fier** **% (n)**	**Rural % (n)**	**Urban % (n)**	**Total % (n)**
Number of interviews	25.9 (183)	74.1 (523)	33.3 (235)	66.7 (471)	100.0 (706)	41.9 (325)	58.1 (451)	53.5 (428)	46.6 (348)	100.0 (776)
Women	53.0 (97)	57.4 (300)	51.9 (122)	58.4 (275)	56.2 (397)	53.5 (174)	58.8 (265)	51.6 (221)	62.6 (218)	57.0 (439)
Urban	52.4 (96)	71.7 (375)	-	-	66.7 (471)	10.9 (104)	35.6 (244)	-	-	46.6 (348)
Average age (SD)	42.3 (25.5)	45.1 (26.8)	39.0 (26.9)	47.3 (25.8)	44.4 (26.5)	50.5 (19.3)	52.2 (21.4)	51.6 (20.3)	51.3 (20.8)	51.5 (20.5)
**Education**										
Never attended school	18.6 (34)	12.7 (66)	17.5 (41)	12.6 (59)	14.2 (100)	6.3 (15)	2.0 (7)	5.7 (21)	0.5 (1)	3.5 (22)
Completed primary school (max. 5 years)	15.3 (28)	10.9 (57)	15.7 (37)	10.2 (48)	12.1 (85)	8.0 (19)	12.2 (42)	12.3 (45)	7.4 (16)	10.8 (61)
Completed compulsory school (max. 8/9 years)	27.9 (51)	30.5 (159)	36.2 (85)	26.7 (125)	29.8 (210)	49.4 (118)	35.6 (122)	44.5 (163)	35.7 (77)	40.2 (240)
Completed high school (12 years)	28.4 (52)	28.2 (147)	19.6 (46)	32.6 (153)	28.3 (199)	26.8 (64)	36.4 (125)	29.8 (109)	37.0 (80)	33.2 (189)
Completed college	3.8 (7)	8.5 (44)	1.3 (3)	10.2 (48)	7.2 (51)	6.7 (16)	13.4 (46)	6.3 (23)	18.1 (39)	11.2 (62)
Other	6.0 (11)	9.2 (48)	9.8 (23)	7.7 (36)	8.4 (59)	2.9 (7)	0.3 (1)	1.4 (5)	1.4 (3)	1.2 (8)
**Occupation**										
Farmer	2.7 (5)	3.8 (20)	8.5 (20)	1.1 (5)	3.6 (25)	3.5 (10)	5.4 (21)	6.7 (26)	1.8 (5)	4.6 (31)
Employed	6.6 (12)	4.4 (23)	2.6 (6)	6.2 (29)	5.0 (35)	9.5 (27)	14.0 (54)	10.3 (40)	14.6 (41)	12.1 (81)
Self-employed business	2.2 (4)	2.7 (14)	2.1 (5)	2.8 (13)	2.6 (18)	2.5 (7)	2.9 (11)	2.1 (8)	3.6 (10)	2.7 (18)
Housewife	18.0 (33)	9.4 (49)	17.0 (40)	8.9 (42)	11.6 (82)	15.9 (45)	15.8 (61)	16.2 (63)	15.4 (43)	15.8 (106)
Government employee, teacher	1.6 (3)	2.9 (15)	0.9 (2)	3.4 (16)	2.6 (18)	1.4 (4)	3.1 (12)	1.8 (7)	3.2 (9)	2.4 (16)
Unemployed	14.8 (27)	11.5 (60)	10.2 (24)	13.4 (63)	12.3 (87)	25.7 (73)	11.9 (46)	20.5 (80)	13.9 (39)	17.8 (119)
Pensioner	27.3 (50)	35.3 (184)	21.3 (50)	39.2 (184)	33.2 (234)	38.0 (108)	42.2 (163)	38.0 (148)	43.9 (123)	40.5 (271)
Other	26.8 (49)	30.1 (157)	37.5 (55)	25.1 (118)	29.2 (206)	3.5 (10)	4.7 (18)	4.6 (18)	3.6 (10)	4.2 (28)
Economic or social aid	21.3 (39)	13.1 (68)	20.0 (47)	12.79 (60)	15.2 (107)	25.6 (83)	22.8 (103)	26.5 (113)	21.0 (73)	23.8 (186)
Ethnic or linguistic minority	1.7 (3)	3.5 (18)	2.6 (6)	3.2 (15)	3.0 (21)	2.5 (8)	5.5 (25)	6.54 (28)	1.4 (5)	4.5 (33)

#### Satisfaction With Health Services

As during the baseline most of the patients in the endline survey had visited a given health facility for 1–3 times in the past 3 months (1–3 times 75%; more than three times 24%). However, a decrease in persons that did not access a given health care facility in the past 3 months, was seen at endline compared with the baseline (baseline, 15%; endline, 0.5%). The decrease was most prominent for Diber and rural areas that moved from 27% and 21% at baseline to 0.5% and 0% at endline (Supplementary Table 1). Visits took place for reasons mostly related to chronic conditions (baseline 40%, endline 49%) followed by conditions not further categorized (baseline 35%, endline 40%) or related to child health (baseline 19%, endline 8%). Less often were the facilities visited for antenatal care (both evaluations 2%) or immunization (baseline 4%, endline 2%) (Supplementary Table 1). This was mostly consistent between base- and endline as well as between Diber vs. Fier and rural vs. urban areas.

When patients were asked, at endline, about their overall satisfaction with the services received on that day, 68% indicated they were very satisfied, 26% were satisfied and 1% were unsatisfied. Approximately 5% indicated overall that they were very unsatisfied with the services received, the proportion of very unsatisfied patients being substantially higher in Diber (10%) than in Fier (1%) and less persons stating to be “very satisfied” (Diber 62% vs. Fier 73%). A difference was also observed between urban and rural facilities with more “very satisfied” patients in urban facilities compared with rural facilities (77.0% vs. 62%) and less patients that were “very unsatisfied” (3% vs. 6%). The proportion of person “unsatisfied” and “satisfied” was similar across groups (1% and 27% for Diber; 0.5% and 25% for Fier; 1% and 31% for Rural; 0.5% and 20% for Urban).

Assessing the patient's satisfaction with different aspects of the health services received, an improvement was seen between base- and endline for three measures, namely (i) patients privacy was ensured (*p* = 0.039); (ii) doctor explained the intake of prescribed medicine (*p* = 0.018); and (iii) doctor asked if patient currently takes prescription (*p* = 0.002) (Table 5). The six other aspects did not notably change between base- and endline.

**Table 5:** Satisfaction with different aspects of health service—exit interviews.

	**Baseline**		**Endline**		
	**Total % (n)**	***N***	**Total % (n)**	***N***	***p*** **-value^c^**
Patient was given the opportunity to explain the health problem	92.5 (653)	706	95.1 (738)	776	0.21
Patients privacy was ensured	90.8 (641)	706	96.6 (750)	776	0.039
Doctor explained the questioning and physical examinations and the health problem^a^	97.0 (518)	534	97.2 (561)	577	0.82
Doctor explained the intake of prescribed medicine^b^	84.5 (354)	419	95.4 (314)	329	0.018
Doctor asked if patient currently takes prescriptions	45.6 (322)	706	63.8 (495)	776	0.002
Patient was given chance to ask questions about the investigation, health problem, and treatment	87.5 (618)	706	90.3 (701)	776	0.40
Doctor listened carefully to patients concerns and questions and gave satisfactory answers	89.8 (634)	706	93.9 (729)	776	0.16
Patient got advice on health problem	82.0 (579)	706	87.2 (677)	776	0.24
Medical doctor was polite during consultation	99.6 (703)	706	98.1 (761)	776	0.12

a *Of those being examined (total baseline n = 534; endline n = 577);*

b *of those being prescribed medicine (baseline n = 419; endline n = 329);*

c *Rao-Scott corrected chi-squared test*.

However, high satisfaction scores were already found at baseline across aspects ranging between 82% and 99% with one exception; i.e., only 46% of patients (endline: 64%) declared that the doctor had asked them whether they are currently taking any other prescriptions (Table 5).

#### Health Insurance and Health Spending

The availability of valid health insurance among patients exiting the health facilities was found to be lower at endline compared to the baseline (84 vs. 90%; *p* = 0.002). At the same time, the number of patients paying for their consultation significantly decreased (*p* < 0.001). Only two patients (0.3%) indicated to have formally paid for services received at endline, while 13 (1%) did so at baseline.

Of those interviewed at endline, 186 declared that they received social or economic aid (*n* = 107 at baseline). Assessing health insurance and health spending measures among recipients of social or economic aid vs. non-recipients, a higher proportion of holders of a valid health insurance card was seen at both baseline and endline among recipients compared with non-recipients (baseline 96% vs. 90%; endline 91% vs. 82%).

The exit interviews also assessed the satisfaction of recipients vs. non-recipients of social or economic aid, with different aspects of the consultations. However, not many differences were observed between the two groups at baseline or endline. The only notable trend found at baseline was that a lower proportion of patients that received social/economic aid felt that their privacy was ensured (85%) compared with those patients that did not receive such aid (92%). This difference, however, did no longer show in the endline assessment. Similarly, at endline, a lower proportion of patients receiving aid, were asked whether they were taking other prescriptions (56.5%) compared with patients not receiving any aid (66%) (Supplementary Table 2). This difference did not show during the baseline assessment.

## Discussion

### Infrastructural Assessment

Functional infrastructure, including physical entities and supporting systems and services, is precondition to an efficient and effective healthcare system (26, 27). A lack of medical care facilities not providing easy access to enough people, or facilities that are operating at sub-standard conditions can deter potential patients from seeking care in a time manner or at all impacting health at individual and population level (11). In the last decade, authorities in Albania have identified infrastructural short falls in their PHC system (8, 9) and have taken steps to address them (5). Overall, the infrastructure situation demonstrated substantial improvements in 2018, compared with the baseline assessment in 2015, with specific improvements seen in the areas of overall cleanliness and availability of basic equipment. Yet, some challenges remain, especially in the area of guidelines and material.

### Clinical Consultations

The clinical observation score and the area-specific sub-scores generally increased at endline, which is likely to be due to the CME and other capacity building measures that were started in 2015. HAP introduced peer groups as a means to establish a viable CME method. Between January 2015 and March 2019 HAP supported an increasing number of peer groups, effectively from eight groups in 2015 to 93 peer groups in 2018. Correspondingly, the number of health care providers attending the groups increased from 68 general practitioners in 2015 to 1'116 health care professionals (i.e., general practitioners, nurses and PHC managers) in 2019. At the end of March 2019, 75% of all family doctors and almost 79% of nurses in both regions were involved in at least one peer group for their continuing education and professional development (28). The median value for clinical observations improved by 19% between 2015 and 2018 representing an increase by almost 5%. Especially, adherence to principles of good clinical practice and physical examination achieved improved and high scores at endline in both regions. With regard to infection prevention and control procedures an improvement was seen in applying proper decontamination procedures in 12% of the medical staff compared with 0% at baseline. Yet, there were some areas that remained at a low score at endline, namely, infection prevention and control and diabetes and hypertension treatment. The same issues were indeed reported from the 2016 baseline assessment in Kosovo (29).

Sub-optimal infection prevention and control has been described in the region previously (30) and has been explained by external factors, namely poor infrastructure, limited resources, insufficient equipment, combined with a lack of guidelines and policies on national level (31). As for masks/gloves, the issue of resource limitation may offer an explanation since national budgets did not allow for a systematic inclusion of personal protective equipment at the PHC level prior to the COVID 19 pandemic. However, the same does not explain the low compliance with hand washing in the current setting. Asking healthcare workers in Pune, India, reasons for non-adherence with hand hygiene were named as unavailability of hand rub at the clinical area, staff shortages and workload pressure (32). Unavailability of a hand rub and outside (time) pressure were also named reasons for non-compliance in a survey with nurses in northwest Ethiopia. Additional reasons included non-accessibility of a sink, damage and irritation to skin, patients getting offended, hands not being dirty and the fact that other colleagues do not practice hand hygiene (33). Some of those reasons may have been also influential to the decision-making of the healthcare workers in Albania and Kosovo. In addition, the processes around clinical observations suffer from poor standardization regarding both the observation and the observer. While the observers in the current study reported on hand washing before a procedure, some doctors strongly emphasized that they would wash their hands at the end of each visit rather than at the beginning of a new consultation.

With regard to diabetes and hypertension care, some improvements were seen between 2015 and 2018; i.e., for diabetes, the category “giving advice, explaining, and instructing” and the overall score was higher at endline compared with the baseline and for the management of hypertension patients, overall improvement was seen as well as for each of the sub-categories. Yet, the overall treatment of diabetes and the practices of conducting examinations for diabetes and hypertension remained at a relatively low level. However, the current observations captures only a part of the ongoing national activities on screening and prevention of NCDs. The MoHPS introduced in 2015 a separate large-scale “check-up” system for NCDs at PHC run by nurses and in collaboration with the private sector, that has carried out annual screenings and testing for a large number of people (482,716 in 2019) (34). This check-up system is part of a timely reaction of the Albanian government to the large increase in NCDs from 1990 to 2010, putting in place different policies and programmes in Albania for NCD control and prevention (4) and prioritizing NCD control and prevention in their National Health Strategy 2016–2020 (5). NCD control and prevention was, e.g., included in the legislation on the health sector such as the Law 10107 of 30.03.2009 “On Health Care in Albania,” Law 9636, of 6.11.2006 “On Health Protection from Tobacco Products,” Law 9518, of 18.4.2006 “On Protection of Minors from Use of Alcohol” and the Food and Nutrition Policy Discussion Paper 2013–2020 (4). Nevertheless, at the healthcare level, the delivery of care for chronic diseases comes with a high workload, especially for the general practitioners that have to screen for risk factors or complications, treat, exchange information of lifestyle modification and ensure follow up, within the few minutes spent with the patient (35).

### Satisfaction With the Health Services

Endline patient satisfaction on PHC services remained largely the same with the exception of three aspects (privacy ensured; doctor explaining intake of prescribed medicine; and doctor asking if patient currently takes prescriptions), that were rated higher at endline compared with the baseline. However, satisfaction ratings among patients are best treated with caution as they might not only reflect the “true” patient satisfaction but may be determined by cultural beliefs (e.g., believe or contrarily lack of trust in authorities), the lack of knowledge and awareness on what actually would constitute good health services and the fear of negative consequences due to high dependencies.

### Health Insurance and Health Spending

The availability of valid health insurance among patients exiting the health facilities was found to be lower at endline compared to the baseline, while at the same time, the number of patients paying for their consultation significantly decreased. The decrease of health insurance holders with subsequent decrease of out-of-pocket payment may be attributed to a substantial policy change between baseline and endline survey. The MoHSP introduced a total gratuity of PHC services in December 2015, regardless of whether the users own an insurance card, implying that patients and consumers do not need a card to get most services free at PHC level. Moreover, the family doctor may refer a person to specialist care. Only few services, e.g., health check for renewal of driving license or documents demonstrating the person ability to work, require payments since the policy was introduced.

### Study Limitations

Medical students acted as observers for the clinical consultations and were trained and received clear instructions prior to data collection. However, the observers were not necessarily the same during the baseline and the endline. In addition, while many observations can be controlled through training of the observers, some subjectivity on the level of the observation remains. While the observers in the current study reported on hand washing before a procedure, some doctors strongly emphasized that they would wash their hands at the end of each visit rather than at the beginning of a new consultation. Moreover, it was also up to the observers to judge on whether a denominator was applicable in a given situation. Mask wearing during a consultation on hypertension may have been perceived as “not applicable” as well as closing of a consultation politely after issuing a prescription or a signature a process not necessarily perceived as real consultation. Equally, the frequency of a patient visiting may influence processes; medical doctors may see the same patient several times a week but take physical measure only once a week. Although, a larger degree of standardization was tried to be introduced, especially during the endline survey, an influence toward the outcomes cannot be excluded.

The experimental design did not allow for linking the data from the clinical observations and the exit interviews. Separating the two processes, however, was necessary to ensure confidentiality and to avoid direct hesitancy and/or a biased response by the patients when having to provide a quality judgement if it can directly be linked to the personal doctor. Two interviewers were therefore present at each site conducting the clinical observations and the exit interviews, respectively. The interview with patients exiting the facility was conducted in the yard or in the large HC corridors, to allow for maximum privacy. Yet, it has been shown previously, that there might still be a larger courtesy bias when interviews take place at the respective public health facility as opposed to home-based surveys with clients reporting substantially higher satisfaction with care in the former (36). On the other hand, immediate post-visit completion of a survey minimizes recall bias.

Also for the health providers—being observed by a third party and/or being aware of the fact that exit interviews are taking place on the same day—a positive bias in their performance cannot be ruled out (Hawthorne effect) (37).

## Conclusion

The changes observed in the 38 PHC facilities in two regions in Albania between 2015 and 2018 were overall positive with improvements seen all three levels assessed, e.g., infrastructure, service provision and patient satisfaction. HAP supported the process applying a multi-strategic health system strengthening approach to a healthcare environment faced with an increasing burden of non-communicable diseases and sup-optimal access to healthcare. To gain overall improvements in the QoC and move toward a more efficient and sustainable health system requires continuous investments in infrastructure alongside interventions at the provider level, i.e., capacity building of health care staff through CME systems and district management capacities, and the user level, by fostering education and behavioral changes at the population level.

## Data Availability Statement

The original contributions presented in the study are included in the article/Supplementary Material, further inquiries can be directed to the corresponding author/s.

## Ethics Statement

The studies involving human participants were reviewed and approved by Ministry of Health and Social Protection that is acting as ethical review body for implementation research in Albania and the Ethics Committee of North-Western and Central Switzerland EKNZ-Ethikkommission Nordwest-und Zentralschweiz. Written informed consent to participate in this study was provided by the participants' legal guardian/next of kin.

## Author Contributions

KW and SK conceptualized the study. AP participated in the data collection, SK analyzed the data. JS drafted the manuscript. AP, KW, and SK critically reviewed and approved the final version of the manuscript. All authors contributed to the article and approved the submitted version.

## Funding

The study has been conducted in the frame of the Health for All Project (Projekti HAP http://www.hap.org.al/) funded by the Swiss Agency for Development and Cooperation (SDC) and implemented by the Swiss Tropical and Public Health Institute.

## Conflict of Interest

The authors declare that the research was conducted in the absence of any commercial or financial relationships that could be construed as a potential conflict of interest.

## Publisher's Note

All claims expressed in this article are solely those of the authors and do not necessarily represent those of their affiliated organizations, or those of the publisher, the editors and the reviewers. Any product that may be evaluated in this article, or claim that may be made by its manufacturer, is not guaranteed or endorsed by the publisher.

## References

[1] Sheiman I Fleck F. Rocky road from the Semashko to a new health model. B World Health Organ. (2013) 91:320–1. 10.2471/BLT.13.030513 23678194 PMC3646351

[2] Nuri B. Health Care Systems in Transition: Albania, Vol. 4. Copenhagen: European Observatory on Health Care Systems (2002).

[3] Albania: Restoring Growth Improving Prosperity. (2017) Available online at: https://www.worldbank.org/en/results/2017/04/17/albania-restoring-growth-and-improving-prosperity (accessed June 22, 2020).

[4] Ministry of Health. National Program on Prevention and Control of NCDs in Albania 2016-2020. Tirana: Ministry of Health (2017).

[5] Ministry of Health. Albania National Health Strategy 2016-2020. Tirana: Ministry of Health (2016).

[6] Lerch M. Internal and international migration across the urban hierarchy in Albania. Popul Res Policy Rev. (2016) 35:851–76. 10.1007/s11113-016-9404-2 27867240 PMC5088220

[7] de Bruijn B Filipi G Nesturi M Galanxhi E. Population ageing: Situation of Elderly People in Albania. Albania: Institute of Statistics (2015).

[8] Arqimandriti M Ivkovic M Naskidashvili I Ekonomi M Skora L Como E. Monitoring of the Primary Health Care System in Albania. Tirana: CFFES Office (2014).

[9] World Health Organization. Primary Health Care in Albania: Rapid Assessment. Geneva: World Health Organization (2018).

[10] Akshija I Dibra A. Hospital doors under pressure; policies and trends in the major tertiary care hospital in Albania. G Chir. (2018) 39:265–71. 30444474

[11] World Health Organization. Primary Health Care on the Road to Universal Health Coverage. In 2019 Monitoring Report. Geneva: WHO (2019).

[12] Hanefeld J Powell-Jackson T Balabanova D. Understanding and measuring quality of care: dealing with complexity. B World Health Organ. (2017) 95:368–74. 10.2471/BLT.16.179309 28479638 PMC5418826

[13] Kruk ME Kelley E Syed SB Tarp F Addison T Akachi Y. Measuring quality of health-care services: what is known and where are the gaps? B World Health Organ. (2017) 95:390. 10.2471/BLT.17.195099 28603302 PMC5463820

[14] Kruk ME Gage AD Joseph NT Danaei G Garcia-Saiso S Salomon JA. Mortality due to low-quality health systems in the universal health coverage era: a systematic analysis of amenable deaths in 137 countries. Lancet. (2018) 392:2170. 10.1016/S0140-6736(18)31668-4 30195398 PMC6238021

[15] Tamburlini G Siupsinskas G Bacci A Quality MNC. Quality of maternal and neonatal care in Albania, Turkmenistan and Kazakhstan: a systematic, standard-based, participatory assessment. PLoS ONE. (2011) 6:e28763. 10.1371/journal.pone.0028763 22216110 PMC3245221

[16] Myftiu S Sulo E Burazeri G Sharka I Shkoza A Sulo G. higher burden of metabolic risk factors and underutilization of therapy among women compared to men might influence a poorer prognosis: a study among acute myocardial infarction patients in Albania, a transitional country in Southeastern Europe. Croat Med J. (2015) 56:542–9. 10.3325/cmj.2015.56.542 26718760 PMC4707925

[17] Peabody JW DeMaria L Smith O Hoth A Dragoti E Luck J. Large-scale evaluation of quality of care in 6 countries of Eastern Europe and Central Asia using clinical performance and value vignettes. Glob Health Sci Pract. (2017) 5:412–29. 10.9745/GHSP-D-17-00044 28963174 PMC5620338

[18] Gabrani J Schindler C Wyss K. Factors associated with the utilisation of primary care services: a cross-sectional study in public and private facilities in Albania. BMJ Open. (2020) 10:e040398. 10.1136/bmjopen-2020-040398 33262191 PMC7709502

[19] Gabrani J Schindler C Wyss K. Health Seeking Behavior Among Adults and Elderly With Chronic Health Condition(s) in Albania. Front Public Health. (2021) 9:616014. 10.3389/fpubh.2021.616014 33796494 PMC8007873

[20] Gabrani J Schindler C Wyss K. Perspectives of public and private primary healthcare users in two regions of albania on non-clinical quality of care. J Prim Care Community Health. (2020) 11:2150132720970350. 10.1177/2150132720970350 33243061 PMC7705804

[21] Swiss Agency for Development Cooperation Swiss Tropical Public Health Institute Terre des Homme Save the Children (2015–2019). Health for All Project. Retreived from: http://www.hap.org.al/en/ (accesesed June 24, 2020).

[22] Donabedian A. The quality of care–how can It be assessed. J Am Med Assoc. (1988) 260:1743–8. 10.1001/jama.1988.03410120089033 3045356

[23] Donabedian A. The 7 pillars of quality. Arch Pathol Lab Med. (1990) 114:1115–8. 2241519

[24] Service Availability Readyness Assessment (SARA). (2015). Available online at: https://www.who.int/healthinfo/systems/sara_related_links/en/ (accessed June 24, 2020).

[25] Mboya D Mshana C Kessy F Alba S Lengeler C Renggli S. Embedding systematic quality assessments in supportive supervision at primary healthcare level: application of an electronic Tool to Improve Quality of Healthcare in Tanzania. BMC Health Serv Res. (2016) 16:578. 10.1186/s12913-016-1809-4 27737679 PMC5064905

[26] Kapologwe NA Meara JG Kengia JT Sonda Y Gwajima D Alidina S. Development and upgrading of public primary healthcare facilities with essential surgical services infrastructure: a strategy towards achieving universal health coverage in Tanzania. BMC Health Serv Res. (2020) 20:218. 10.1186/s12913-020-5057-2 32183797 PMC7076948

[27] Hospitals: Infrastructure Technologies. (2020). Available online at: https://www.who.int/hospitals/infrastructure-and-technologies/en/ (accessed July 15, 2020).

[28] Nuri B Wyss K. Annual Progress Report January 2018 to March 2019 and Final Phase Report 2015-2019. Tirana: Health for All Project (2019).

[29] Primary Health Care in Kosovo. Quality of Care Study 2016 Summary Report: Accessible Quality Healthcare. Kosovo: Primary Health Care (2016).

[30] Licker M Baditoiu L Lungeanu D Dobrevska R Szilagy E Raka L. Infection control capacity building in European countries with limited resources: issues and priorities. J Hosp Infect. (2017) 96:85–8. 10.1016/j.jhin.2016.12.024 28153557

[31] Raka L Mulliqi-Osmani G. Infection Control in Developing World. London: IntechOpen (2014)

[32] Chavali S Menon V Shukla U. Hand hygiene compliance among healthcare workers in an accredited tertiary care hospital. Indian J Crit Care Med. (2014) 18:689–93. 10.4103/0972-5229.142179 25316980 PMC4195200

[33] Engdaw GT Gebrehiwot M Andualem Z. Hand hygiene compliance and associated factors among health care providers in Central Gondar zone public primary hospitals, Northwest Ethiopia. Antimicrob Resist Infect Control. (2019) 8:190. 10.1186/s13756-019-0634-z 31788237 PMC6880540

[34] Albanian Health Insurance Fund. Annual Report 2019 (“Raporti Vjetor 2019”). Tirana: Albanian Health Insurance Fund (2019).

[35] Lall D Engel N Devadasan N Horstman K Criel B. Challenges in primary care for diabetes and hypertension: an observational study of the Kolar district in rural India. BMC Health Serv Res. (2019) 19:44. 10.1186/s12913-019-3876-9 30658641 PMC6339380

[36] Hameed W Ishaque M Gul X Siddiqui JU Hussain S Hussain W. Does courtesy bias affect how clients report on objective and subjective measures of family planning service quality? A comparison between facility- and home-based interviews. Open Access J Contracept. (2017) 9:33–43. 10.2147/OAJC.S153443 29760573 PMC5937485

[37] Leonard KL Masatu MC. Using the Hawthorne effect to examine the gap between a doctor's best possible practice and actual performance. J Dev Econ. (2010) 93:226–34. 10.1016/j.jdeveco.2009.11.001

