# Supplementary material for: Assessing the Quality of Care at Primary Health Care Level in Two Pilot Regions of Albania
Source: Front Public Health. 2021 Dec 22;9:747689. doi: 10.3389/fpubh.2021.747689 (PMC8727515; doi:10.3389/fpubh.2021.747689)
Supplement: Supplementary file 1 [file Table_1.DOCX]

**Table S1** Frequency and reason of visit prior to exit interview

|  | **Baseline** | | | | | **Endline** | | | | |
| --- | --- | --- | --- | --- | --- | --- | --- | --- | --- | --- |
|  | **Diber %**  **(n= 183)** | **Fier %**  **(n= 523)** | **Rural % (n=235)** | **Urban %**  **(n= 471)** | **Total %**  **(n=706)** | **Diber %**  **(n= 325)** | **Fier %**  **(n= 451)** | **Rural % (n=428)** | **Urban %**  **(n=348)** | **Total %**  **(n=776)** |
| Excluding today: How often did you over the past 3 months access this HC? | | | | | | | | | | |
| Did not access this HC in the past 3 months | 26.8% | 10.3% | 21.3% | 11.3% | 14.6% | 0.3% | 0.7% | 0.0% | 1.2% | 0.5% |
| 1-3 times | 46.5% | 58.7% | 52.8% | 56.9% | 55.5% | 72.0% | 67.4% | 75.5% | 74.4% | 75.4% |
| More than 3 times | 26.8% | 31.0% | 26.0% | 31.9% | 29.9% | 27.7% | 31.9% | 24.5% | 24.4% | 24.0% |
| What was the reason for your consultation today? | | | | | | | | | | |
| Chronic condition | 31.2% | 42.6% | 30.2% | 44.4% | 39.6% | 39.7% | 53.9% | 41.8% | 55.5% | 49.0% |
| Antenatal care | 0.6% | 2.1% | 2.6% | 1.3% | 1.7% | 0.9% | 2.2% | 0.7% | 2.9% | 1.8% |
| Child health | 19.7% | 19.1% | 23.4% | 17.2% | 19.3% | 9.2% | 6.7% | 5.8% | 10.2% | 7.5% |
| Immunisation | 5.5% | 3.6% | 7.2% | 2.6% | 4.1% | 1.9% | 2.2% | 2.6% | 1.4% | 2.1% |
| Other | 43.2% | 32.5% | 36.6% | 34.6% | 35.3% | 48.3% | 35.0% | 49.2% | 30.2% | 39.6% |

**Table S2** Satisfaction with different aspects of health service - exit interviews among persons receiving social or economic aid

|  | **Baseline** | | **Endline** | |
| --- | --- | --- | --- | --- |
|  | **Not receiving social or economic aid %**  **(n=597)** | **Receiving social or economic aid %**  **(n=107)** | **Not receiving social or economic aid %**  **(n=589)** | **Receiving social or economic aid %**  **(n=186)** |
| … patient was given the opportunity to explain the health problem | 92.1% | 94.4% | 95.4% | 94.1% |
| …patients privacy was ensured | 91.8% | 85.1% | 97.1% | 95.2% |
| …doctor explained the questioning and physical examinations and the health problem | 97.5%  (n=435 of 446) | 94.2%  (n=81 of 86) | 97.0%  (n=426 of 439) | 97.8% (n=134 of 137) |
| … doctor explained the intake of prescribed medicine | 84.5%  (n=299 of 354) | 84.4%  (n=54 of 64) | 95.3% (n=244 of 256) | 95.9% (n=70 of 73) |
| …. doctor asked if patient currently takes prescriptions | 45.6% | 44.9% | 66.2% | 56.5% |
| … patient was given chance to ask questions about the investigation, health problem and treatment | 87.4% | 88.8% | 89.8% | 91.9% |
| … doctor listened carefully to patients concerns and questions and gave satisfactory answers | 89.5% | 91.6% | 93.2% | 96.2% |
| … patient got advice on health problem | 81.6% | 84.1% | 88.1% | 84.4% |
| … medical doctor was polite during consultation | 99.5% | 100.0% | 98.1% | 97.9% |
